# Deletion of Sphingosine 1‐Phosphate receptor 1 in cardiomyocytes during development leads to abnormal ventricular conduction and fibrosis

**DOI:** 10.14814/phy2.15060

**Published:** 2021-10-07

**Authors:** Ryan Jorgensen, Meghna Katta, Jayne Wolfe, Desiree F. Leach, Bianca Lavelle, Jerold Chun, Lisa D. Wilsbacher

**Affiliations:** ^1^ Feinberg Cardiovascular and Renal Research Institute Northwestern University Feinberg School of Medicine Chicago Illinois USA; ^2^ Department of Medicine Northwestern University Feinberg School of Medicine Chicago Illinois USA; ^3^ Department of Pharmacology Northwestern University Feinberg School of Medicine Chicago Illinois USA; ^4^ Sanford Burnham Prebys Medical Discovery Institute La Jolla California USA

**Keywords:** cardiac conduction, fibrosis, S1P, S1P_1_, sphingosine 1‐Phosphate receptor 1

## Abstract

Sphingosine 1‐Phosphate receptor 1 (S1P_1_, encoded by *S1pr1*) is a G protein‐coupled receptor that signals in multiple cell types including endothelial cells and cardiomyocytes. Cardiomyocyte‐specific deletion of *S1pr1* during mouse development leads to ventricular noncompaction, with 44% of mutant mice surviving to adulthood. Adult survivors of embryonic cardiomyocyte *S1pr1* deletion showed cardiac hypertrabeculation consistent with ventricular noncompaction. Surprisingly, systolic function in mutant mice was preserved through at least 1 year of age. Cardiac conduction was abnormal in cardiomyocyte *S1pr1* mutant mice, with prolonged QRS intervals in mutants as compared with littermate control mice. Immunostaining of hearts from *S1pr1* mutant embryos displayed a zone of intermediate Connexin 40 (Cx40) expression in the trabecular myocardium. However, we observed no significant differences in Cx40 and Connexin 43 immunostaining in hearts from adult survivors of embryonic cardiomyocyte *S1pr1* deletion, which suggests normalized development of the ventricular conduction system in mutant mice. By contrast, the adult survivors of embryonic cardiomyocyte *S1pr1* deletion showed increased cardiac fibrosis as compared with littermate controls. These results demonstrate that ventricular hypertrabeculation caused by embryonic deletion of cardiomyocyte *S1pr1* correlates with cardiac fibrosis, which contributes to abnormal ventricular conduction. These results also reveal conduction abnormalities in the setting of hypertrabeculation with normal systolic function, which may be of clinical relevance in humans with ventricular hypertrabeculation.

## Graphical abstract

In adult mice that survived embryonic deletion of *S1pr1* in cardiomyocytes, we observed cardiac hypertrabeculation and conduction abnormalities with prolonged QRS. The conduction system marker Connexin 40 (Cx40) displayed an abnormal expression pattern in *Mlc2a* ^Cre/+^; *S1pr1* ^f/−^ mutant embryos. However, Cx40 and contactin‐2 expression in adult mice did not differ from littermate controls, which suggests normalized development and maintenance of the ventricular conduction system. Finally, cardiomyocyte *S1pr1* mutant mice demonstrated increased cardiac fibrosis that correlated with QRS prolongation.

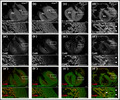

## 1 INTRODUCTION

Sphingosine1‐Phosphate (S1P) is a bioactive lipid that acts via one of the five G protein‐coupled receptors named S1P_1_ρS1P_5_ (Chun et al., 2019; Kihara et al., 2014). *S1pr1*, the gene that encodes S1P_1_, is highly expressed in endothelial cells, lymphocytes, neuronal cells, and cardiomyocytes (Choi et al., 2011; Clay et al., 2016; Liu et al., 2000; Matloubian et al., 2004). Global and endothelial‐specific knockout of *S1pr1* in mice causes abnormal vessel development and death at midgestation due to defective sprouting angiogenesis (Allende et al., 2003; Gaengel et al., 2012; Jung et al., 2012; Liu et al., 2000). We demonstrated that cardiomyocyte‐specific loss of *S1pr1* during development led to ventricular septal defects and ventricular noncompaction (Clay et al., 2016). These structural abnormalities caused perinatal death in 68% of mutant mice; however, the remaining *S1pr1* cardiomyocyte mutant mice survived to adulthood (Clay et al., 2016).

The ventricular conduction system (VCS) comprises bundle of His, the left and right bundle branches, and Purkinje fibers; this subset of cardiomyocytes is distinct from the working myocardium that supports contraction (Dobrzynski et al., 2013; Miquerol et al., 2010; Sedmera, 2011). Gap junctions between cardiomyocytes contribute to the rapid conduction of action potentials both within the VCS and in working myocardium. Purkinje fibers relay the action potential from the proximal VCS to the myocardium and coordinate simultaneous contraction of the ventricular working myocardium. Connexons, made up of connexin protein subunits, create the pores between cardiomyocytes at gap junctions (Verheule & Kaese, 2013). Cardiomyocytes within the VCS highly express Connexin 40 (Cx40), while both VCS and working myocardium express Connexin 43 (Cx43) (Gourdie et al., 1993; Severs et al., 2008). Abnormal expression of either Cx40 or Cx43 has been reported in patients with ventricular conduction disease, and mouse models support roles for both connexins in coordinating normal ventricular conduction (Akar et al., 2004; Gourdie et al., 1993; Mueller et al., 2011; Sedmera et al., 2016; Tang et al., 2013). In humans, left ventricular noncompaction cardiomyopathy is often associated with ventricular arrhythmias, but mechanisms remain unclear (Oechslin & Jenni, 2011; Wilsbacher & McNally, 2016). Of note, Purkinje fibers originate from trabecular myocardium, and how the persistence of trabecular myocardium affects conduction through the VCS and working myocardium is not well understood. Additionally, structural abnormalities of the working myocardium, such as cardiac fibrosis, can cause arrhythmias due to disrupted conduction pathways and late depolarization within regions of fibrosis (Nazarian et al., 2010; Nguyen et al., 1988).

Here, we describe the phenotype of adult mice that survived cardiomyocyte‐specific excision of *S1pr1* during development. All mutant mice displayed cardiac hypertrabeculation consistent with ventricular noncompaction, which established this genetic approach as a model system for human left ventricular noncompaction. Mutant mice also showed prolonged QRS duration, a sign of aberrant ventricular conduction. Because the VCS is derived from trabecular myocardium, we asked whether the conduction system develops normally in hearts with abnormally persistent trabeculation.

## 2 METHODS

### 2.1 Nomenclature

Sphingosine 1‐Phosphate receptor 1 protein is called S1P_1_ by International Union of Basic and Clinical Pharmacology nomenclature. *S1pr1* is the mouse gene that encodes S1P_1_ (Kihara et al., 2014).

### 2.2 Mouse strains

**FIGURE 1:**
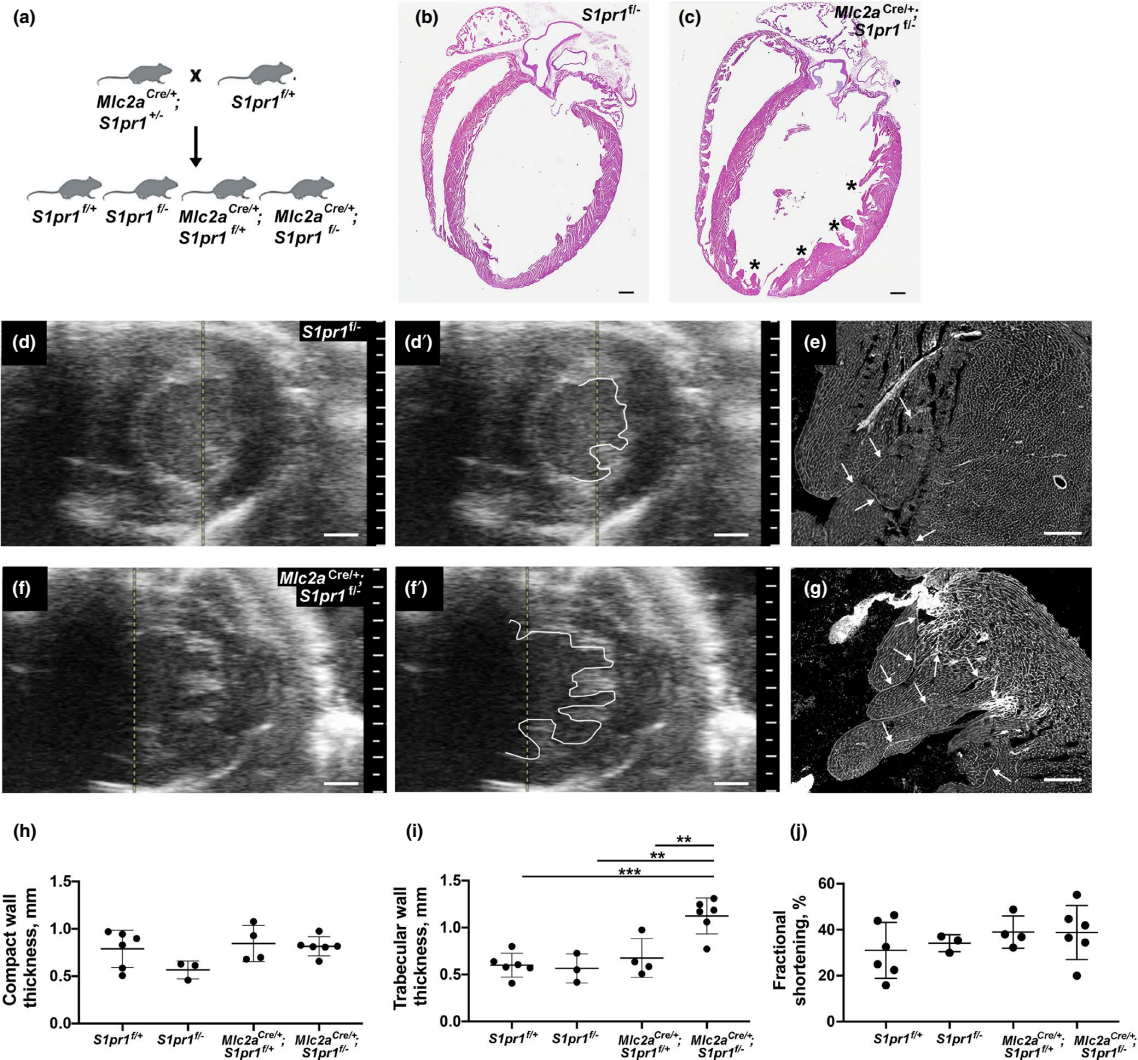
Cardiomyocyte‐specific excision of S1pr1 during development leads to abnormal cardiac structure in adult mice. (a) Breeding strategy. (b, c) Representative H&E staining in 1‐year‐old *S1pr1* ^f/−^ control (b) and *Mlc2a* ^Cre/+^; *S1pr1* ^f/−^ mutant (c) mice. Asterisks indicate hypertrabeculation at regions of thin compact wall in the mutant heart. (d–g) Representative images from 12‐month‐old *S1pr1* ^f/−^ control (d, d′, e) and *Mlc2a* ^Cre/+^; *S1pr1* ^f/−^ mutant (f, f′, g) mice. (d, f) Short‐axis echocardiography images captured in diastole. (d′, f′) Same images as (d and f), respectively, with white line highlighting the endocardial border. Note hypertrabeculation in (f) and (f′). Scale bar, 1 mm. (e, g) Wheat germ agglutinin (WGA)‐Alexa 488 conjugate staining of the sections from the same hearts depicted in (d) and (f), respectively. Arrows highlight trabeculae lined by endocardium. Note increased number and longer length of trabeculae in the *Mlc2a* ^Cre/+^; *S1pr1* ^f/−^ mutant. Scale bar, 200 µm. (h) Compact wall thickness in 6‐ to 12‐month‐old mice from the four genotypes generated by the breeding strategy. No significant differences were observed. (i) Trabecular wall thickness in mice from the four genotypes. **p < 0.01 and ***p < 0.001 by one‐way ANOVA with Bonferroni post‐hoc test for multiple comparisons. (j) Fractional shortening in mice from the four genotypes revealed no significant differences. *n* = 3–6 mice per genotype

Richard Proia (National Institute of Diabetes and Digestive and Kidney Diseases) provided *S1pr1* global heterozygous knockout mice (*S1pr1^tm2Rlp^*, designated as *S1pr1* ^+/−^ mice) (Liu et al., 2000). Dr. Jerold Chun provided mice carrying a floxed *S1pr1* allele (*S1pr1^tm1Jch^*; designated as *S1pr1* ^f/f^) (Choi et al., 2011). Dr. Xu Peng (Scott & White Memorial Hospital) provided *Mlc2a*‐Cre knockin mice (*Myl7^tm1(cre)Krc^*, designated as *Mlc2a* ^Cre/+^) (Wettschureck et al., 2001). Mice with conditional deletion of *S1pr1* were generated as previously described (Clay et al., 2016). We used *Mlc2a* ^Cre/+^ rather than *Mlc2v* ^Cre/+^ due to more efficient Cre‐mediated excision in embryonic ventricular cardiomyocytes using *Mlc2a* ^Cre/+^ (Clay et al., 2016; Peng et al., 2008). Briefly, to avoid germline excision in the setting of exogenous Cre expression during development, we maintained *Mlc2a* ^Cre/+^ on the *S1pr1* ^+/−^ background. Conditional *S1pr1* mutant mice were generated in crosses of *S1pr1* ^f/f^ females (mixed C57BL6/J and 129SvJ strain background) with *Mlc2a* ^Cre/+^; *S1pr1* ^+/−^ males (C57BL6/J strain). This breeding strategy created *Mlc2a* ^Cre/+^; *S1pr1* ^f/−^ mutant mice that lack *S1pr1* in cardiomyocytes, as well as *S1pr1* ^f/+^, *S1pr1* ^f/−^, and *Mlc2a* ^Cre/+^; *S1pr1* ^f/+^ littermate controls (Figure 1a). Dr. Shaun Coughlin (University of California, San Francisco) provided sphingosine kinase 1 global knockout mice (*Sphk1* ^tm1Cgh^, designated as *Sphk1* ^−/−^), conditional sphingosine kinase 1 mice (*Sphk1* ^tm2Cgh^, designated as *Sphk1* ^f/f^), and sphingosine kinase 2 global knockout mice (*Sphk2* ^tm1.1Cgh^, designated as *Sphk2* ^−/−^). For *Sphk1*; *Sphk2* cardiomyocyte deletion studies, we maintained *Mlc2a* ^Cre/+^ on the *Sphk1* ^+/−^; *Sphk2* ^−/−^ background. *Mlc2a* ^Cre/+^; *Sphk1* ^+/−^; *Sphk2* ^−/−^ males were bred with *Sphk1* ^f/f^; *Sphk2* ^−/−^ females to generate *Mlc2a* ^Cre/+^; *Sphk1* ^f/−^; *Sphk2* ^−/−^ with deletion of sphingosine kinases in cardiomyocytes (Figure S1a).

### 2.3 Mouse procedures

All mouse procedures were approved by the Institutional Animal Care and Use Committee at Northwestern University. Both male and female mice were used between 6 and 12 months of age in adult mouse echocardiographic and electrocardiographic studies.

### 2.4 Embryo collection

The morning of vaginal plug was defined as 0.5 days post coitus (dpc). Pregnant females were euthanized at 15.5 dpc. Embryos were removed to ice‐cold PBS, dissected away from the placenta, and snap frozen in OCT using liquid nitrogen‐cooled isopentane. For a subset of embryos, the heart was dissected out and frozen in OCT. In all cases, a portion of the embryo was removed for genotyping.

### 2.5 Echocardiography

Adult mouse echocardiograms were recorded using a Vevo2100 echocardiography platform with a 550 MHz solid‐state probe (FujiFilm). Mice were anesthetized using 2% isoflurane, and anesthesia was maintained using 1% isoflurane during collection of echocardiograms. Mouse heart rate was maintained between 400 and 600 (bpm). Recordings were taken from both parasternal long‐axis and short‐axis views. Left ventricular wall thickness and internal dimensions during both systole and diastole were measured approximately midway through the chamber. Fractional shortening was calculated using measurement from three beats per recording. Compact and trabecular wall thickness were measured in diastole using Fiji (Schindelin et al., 2012): trabeculae were measured as projections into the ventricular space, and the remaining myocardium was measured as compact myocardium.

### 2.6 Electrocardiography

Adult mouse electrocardiograms (ECGs) were recorded using the ECGenie platform (Mouse Specifics, Inc.). Mice were acclimated to the apparatus, then placed on the electrode pad for approximately 20 minutes. Mice were not anesthetized during the recording of ECGs. A consistent and representative subsection of 10–20 cardiac cycles of the ECG recordings was analyzed using the EzCG Signal Analysis Software.

### 2.7 Adult heart collection

Mice were anesthetized using 4% isoflurane. Once anesthetized, thoracotomy was performed, the heart was fully exposed, the inferior vena cava was severed to release blood, and 3–5 ml of cardioplegia (128 mM sodium chloride, 2.5 mM magnesium sulfate, 15 mM potassium chloride, 10 mM glucose, 0.62 mM sodium dihydrate phosphate, 10 mM HEPES, 1mM calcium chloride dihydrate) solution was perfused into the left ventricle (LV). The heart was removed, placed into an aluminum ladle, and snap frozen using liquid nitrogen. Frozen hearts were stored at −80°C until cryosectioning. For samples that underwent paraformaldehyde fixation, hearts were perfused with 4% paraformaldehyde in cardioplegia, dissected out of the thorax, fixed overnight in 4% paraformaldehyde in phosphate‐buffered saline, and then processed for paraffin embedding.

### 2.8 Sectioning and immunofluorescence staining

Microtome sections of 6 µm thickness and cryosections of 10 µm thickness were collected as frontal sections of the hearts. Sections selected for staining contained both ventricles and the mitral valve in order to include the VCS. For hematoxylin and eosin (H&E) staining, sections were rehydrated and stained using standard procedures. For wheat germ agglutinin (WGA) staining, sections were fixed using 4% paraformaldehyde and incubated with WGA Alexa Fluor 488 conjugate (ThermoFisher #W11261) at 5 µg/ml for 10 minutes. Immunostaining was performed as previously described (Clay et al., 2016; Wilsbacher & Coughlin, 2015). Briefly, sections were fixed using acetone and permeabilized using Triton X‐100. Sections were blocked in Western Blocking Reagent (Roche #11921673001) and a second blocking step with goat anti‐mouse IgG (H + L) monovalent Fab fragment (Jackson ImmunoResearch #115‐007–003) was used to reduce background in the setting of mouse monoclonal primary antibodies. Sections were incubated with primary antibody overnight at 4°C. Primary antibodies included anti‐α‐actinin (sarcomeric) (1:2000, Sigma #A7811), anti‐Connexin‐40 (1:1500, Alpha Diagnostic #Cx40‐A), anti‐Connexin‐43 (1:1500, Sigma #C6219), anti‐N‐Cadherin (1:300, Abcam #ab18203), and anti‐Collagen1a1 (1:500, Abcam #ab34710). Secondary antibodies included goat anti‐mouse Alexa Fluor 488 (1:2000, Life Technologies #A‐11001) and goat anti‐rabbit Alexa Fluor 568 (1:1000, Life Technologies #A‐11011). Nuclei were stained using Hoechst (1:2000, Life Technologies #H3570). Slides were post‐fixed using 1% PFA and mounted using Prolong Diamond Anti‐Fade (ThermoFisher #P36961).

### 2.9 Microscopy

All imaging and analyses were performed blind to genotype. Imaging was performed using a Zeiss Axio Observer epifluorescence microscope with Apotome‐2 for optical sectioning and ZEN imaging software, or with a Keyence BZ‐X810 digital brightfield‐epifluorescence microscope and BZ‐X800 imaging software. Within each experiment, images were taken using the same exposure times per channel for each sample. Composite images of entire sections were taken using the tiling function within ZEN and BZ‐X800. Images were processed using Fiji (Schindelin et al., 2012); within each experiment, brightness and contrast were set to the same values for each image. Percent area of fibrosis was determined using composite images of entire WGA‐stained sections. For each section, areas of interstitial WGA Alexa Fluor 488 signal within the left ventricular myocardium were measured blind to genotype; the ratio of the sum of those areas to the total LV myocardium area was multiplied by 100 to calculate the percent area of fibrosis. WGA staining in perivascular regions, valves and papillary muscle tips connecting to the mitral valve were not included in the interstitial fibrosis measurements.

### 2.10 Immunoblotting

Ventricular tissue was collected at 7 weeks of age and homogenized in lysis buffer (150 mM sodium chloride, 1.0% Triton X‐100, 0.5% sodium deoxycholate, 0.1% Sodium dodecyl sulfate, 50 mM Tris, pH 8.0) using silica beads and a homogenizer (BioSpec). Lysates were measured using the Pierce BCA assay kit (Thermo Scientific #23225) and normalized to 10 µg total protein. Lysates were separated using polyacrylamide gel electrophoresis and gel was transferred to nitrocellulose (NC) membranes. Membranes were blocked in 5% BSA prepared in 1x TBS‐T. After blocking, NC membranes were incubated with primary antibodies Cx40 (1:1000, Cx40‐A, Alpha Diagnostic Intl Inc.), Connexin 43 (1:1000, Cx43, Sigma C6219), or Contactin 2 (1:1000, R&D Systems, AF4439). Membranes were incubated with secondary antibody goat anti‐rabbit IgG‐HRP (1:1000, Santa Cruz Biotechnology sc‐2004) or donkey anti‐goat IgG‐HRP (1:1000, Santa Cruz Biotechnology sc‐2020). Signals were detected using SuperSignal West Pico PLUS Chemiluminescent Substrate (Thermo Scientific #34577). Total protein was determined using Memcode Reversible Protein Stain Kit (Thermo Scientific #24580).

### 2.11 Statistics

All measurements were done blind to genotype. Differences were assessed using one‐way ANOVA, and post‐hoc individual comparisons were performed using the Bonferroni test. Correlation between QRS length and percent fibrosis was assessed using Pearson correlation coefficients. Genotype distributions were assessed using the chi‐square goodness of fit test. All statistical analyses were performed using GraphPad software. *p* < 0.05 was considered statistically significant.

## 3 RESULTS

### 3.1 Cardiomyocyte‐specific excision of *S1pr1* during development leads to abnormal cardiac structure in adult mice

We generated mice with cardiomyocyte‐specific deletion of *S1pr1* during embryonic development using a Cre recombinase driven by the promoter for *Myl7* (encodes myosin light chain 2a or Mlc2a, hereafter called *Mlc2a* ^Cre/+^). To avoid germline excision in the setting of exogenous Cre expression during development, we maintained *Mlc2a* ^Cre/+^ on the *S1pr1* ^+/−^ background. Conditional *S1pr1* mutant mice were generated in crosses of *S1pr1* ^f/f^ females with *Mlc2a* ^Cre/+^; *S1pr1* ^+/−^ males. This breeding strategy created *Mlc2a* ^Cre/+^; *S1pr1* ^f/−^ mutant mice that lack *S1pr1* in cardiomyocytes, as well as *S1pr1* ^f/+^, *S1pr1* ^f/−^, and *Mlc2a* ^Cre/+^; *S1pr1* ^f/+^ littermate controls (Figure 1a). We previously reported that *Mlc2a* ^Cre/+^; *S1pr1* ^f/−^ mice show cardiac structural abnormalities including ventricular noncompaction; in that study, the majority (68%) of mutant mice died in the perinatal period, but the surviving cardiomyocyte *S1pr1* mutant animals had a normal lifespan of 18 months or longer (Clay et al., 2016). Subsequent litters used in this study again demonstrated fewer *Mlc2a* ^Cre/+^; *S1pr1* ^f/−^ mice at weaning than expected by Mendelian distribution, but a higher percentage (44%) survived to weaning (Table S1). Echocardiography as well as histology using H&E and WGA staining revealed that hearts from control littermates showed normal left ventricular wall morphology (Figure 1b,d,e); by contrast, hearts from *Mlc2a* ^Cre/+^; *S1pr1* ^f/−^ mutant mice displayed persistent trabeculation consistent with ventricular noncompaction (Figure 1c,f,g). Left ventricular compact wall thickness was not different between genotypes, but trabecular wall measurements were significantly greater in *Mlc2a* ^Cre/+^; *S1pr1* ^f/−^ mutant mice as compared with controls (Figure 1h,i; Table S2). Unexpectedly, left ventricular systolic function remained normal in cardiomyocyte *S1pr1* mutant mice (Figure 1j; Table S2). None of the adult *Mlc2a* ^Cre/+^; *S1pr1* ^f/−^ mutant mice had a ventricular septal defect, but all demonstrated abnormal trabeculation.

The source of S1P ligand that activates cardiomyocyte S1P_1_ is not definitively known and potentially includes endocrine, paracrine, and autocrine sources. Mammals carry two sphingosine kinase genes, sphingosine kinase‐1 (*Sphk1*), and sphingosine kinase‐2 (*Sphk2*); their protein products catalyze phosphorylation of sphingosine to S1P (Pappu et al., 2007; Spiegel & Milstien, 2003). To assess whether S1P produced within cardiomyocytes is necessary for the hypertrabeculation phenotype, we generated mice with cardiomyocyte‐specific deletion of *Sphk1* and *Sphk2*. For these studies, we maintained *Mlc2a* ^Cre/+^ on the *Sphk1* ^+/−^; *Sphk2* ^−/−^background. *Mlc2a* ^Cre/+^; *Sphk1* ^+/−^; *Sphk2* ^−/−^ males were bred with *Sphk1* ^f/f^; *Sphk2* ^−/−^ females to generate *Mlc2a* ^Cre/+^; *Sphk1* ^f/−^; *Sphk2* ^−/−^ mice with deletion of sphingosine kinases in cardiomyocytes (Figure S1a). At weaning, we found the expected distribution of all genotypes including *Mlc2a* ^Cre/+^; *Sphk1* ^f/−^; *Sphk2* ^−/−^ (Table S3). Furthermore, we did not observe differences in cardiac structure by echocardiography in *Mlc2a* ^Cre/+^; *Sphk1* ^f/−^; *Sphk2* ^−/−^ versus littermate control mice (Figure S1b,c). These results argue against autocrine or cardiomyocyte cell‐autonomous roles for S1P in cardiac development; rather, these results indicate that either endocrine or paracrine sources of S1P act on the S1P_1_ receptor in cardiomyocytes.

### 3.2 Cardiomyocyte *S1pr1* mutants display abnormal ventricular conduction

**FIGURE 2:**
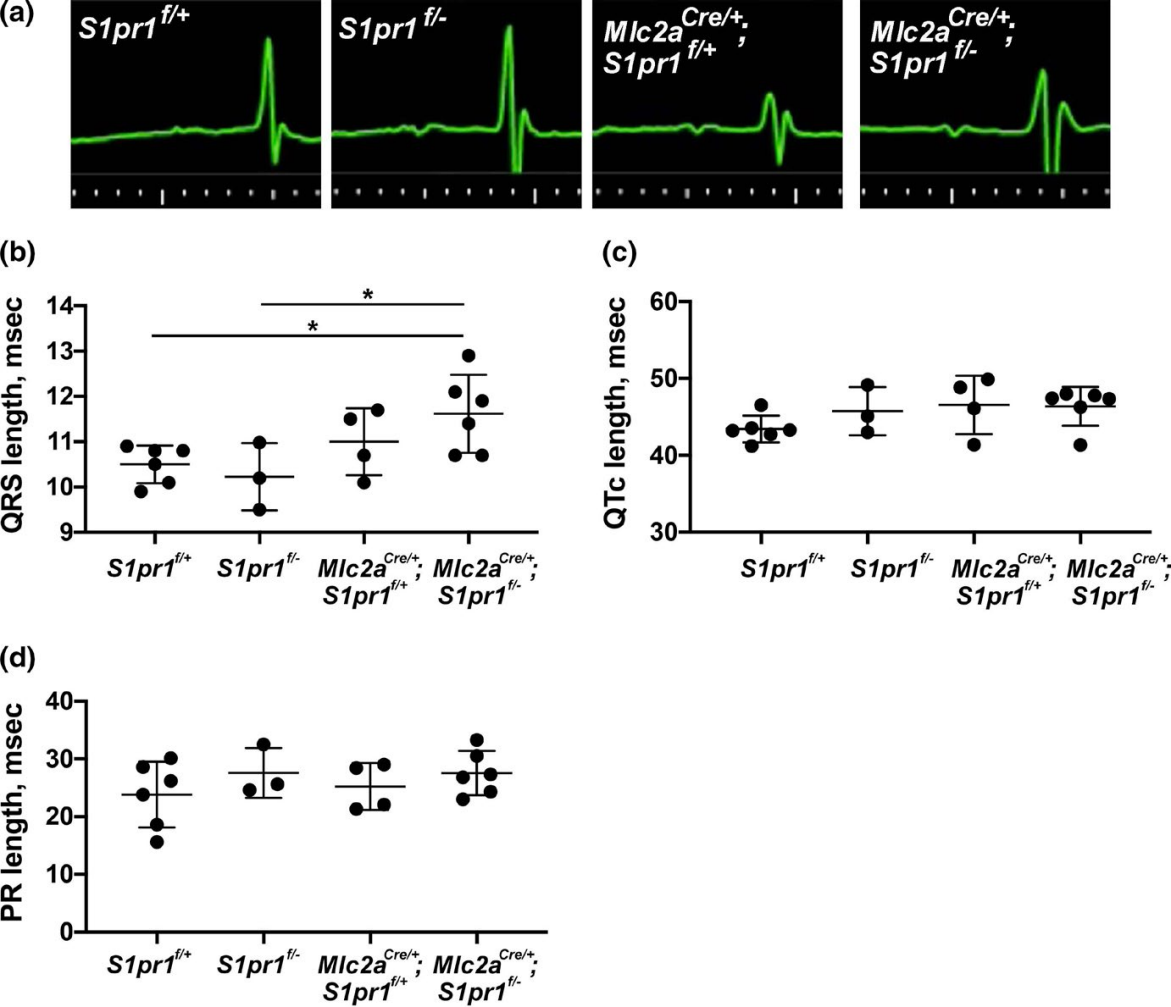
Cardiomyocyte S1pr1 mutants display abnormal ventricular conduction. (a) Representative electrocardiography (ECG) tracings in green obtained from 6‐month‐old mice during echocardiography. Genotypes are noted for each tracing. (b) QRS length, (c) corrected QT (QTc) length, and (d) PR length from mice of each genotype as measured in 6‐ to 12‐month‐old awake mice using the ECGenie system. *n* = 3–6 mice per genotype. *p < 0.05 by one‐way ANOVA with Bonferroni post‐hoc test for multiple comparisons

During echocardiography, we frequently noted wide QRS intervals on the ECG tracings in mice that also showed features of hypertrabeculation on echocardiography imaging (Figure 2a). The QRS interval reflects ventricular depolarization, and multiple forms of cardiomyopathy are associated with prolonged QRS interval (Brenyo & Zareba, 2011). To further investigate whether cardiomyocyte‐specific *S1pr1* mutant mice had prolonged QRS, and to determine whether this feature was dependent or independent of isoflurane anesthesia, we collected ECG data in awake mice using the ECGenie platform. We observed no difference in QRS duration between *S1pr1* ^f/+^ and *S1pr1* ^f/−^ mice, intermediate QRS prolongation in *Mlc2a^Cre^* ^/+^; *S1pr1^f^* ^/+^ mice, and the longest QRS duration in *Mlc2a^Cre^* ^/+^; *S1pr1^f^* ^/−^ mutant mice (Figure 2b). QRS duration was significantly longer in *Mlc2a^Cre^* ^/+^; *S1pr1^f^* ^/−^ mutant mice as compared with *S1pr1* ^f/+^ and *S1pr1* ^f/−^ mice, but the intermediate QRS duration in *Mlc2a^Cre^* ^/+^; *S1pr1^f^* ^/+^ control mice was not significantly different from any other group. These results suggest a potential interaction between *Mlc2a* haploinsufficiency and *S1pr1* dosage. The lack of difference in QRS duration between *S1pr1* ^f/+^ mice and *S1pr1* ^f/−^ mice indicates that heterozygosity for *S1pr1* alone does not drive changes in cardiac conduction. However, these data suggest that the loss one copy of *Mlc2a*, the presence of the Cre recombinase, or both may contribute to ventricular conduction abnormalities, and that the combination of these genetic changes in addition to loss of *S1pr1* in cardiomyocytes further compounds ventricular conduction abnormalities.

Given the genotype‐specific differences in ventricular depolarization, we looked for changes in other portions cardiac conduction pathway. We observed no differences in the corrected QT (QTc) interval, which reflects ventricular repolarization (Figure 2c). We also found no genotype‐specific differences in the PR interval, which reflects conduction from the atria to the ventricles through the atrioventricular node (Figure 2d). Overall, these results indicate that cardiomyocyte‐specific *S1pr1* deletion specifically affects ventricular depolarization during the cardiac conduction cycle.

### 3.3 VCS **development after embryonic cardiomyocyte *S1pr1* deletion**

**FIGURE 3:**
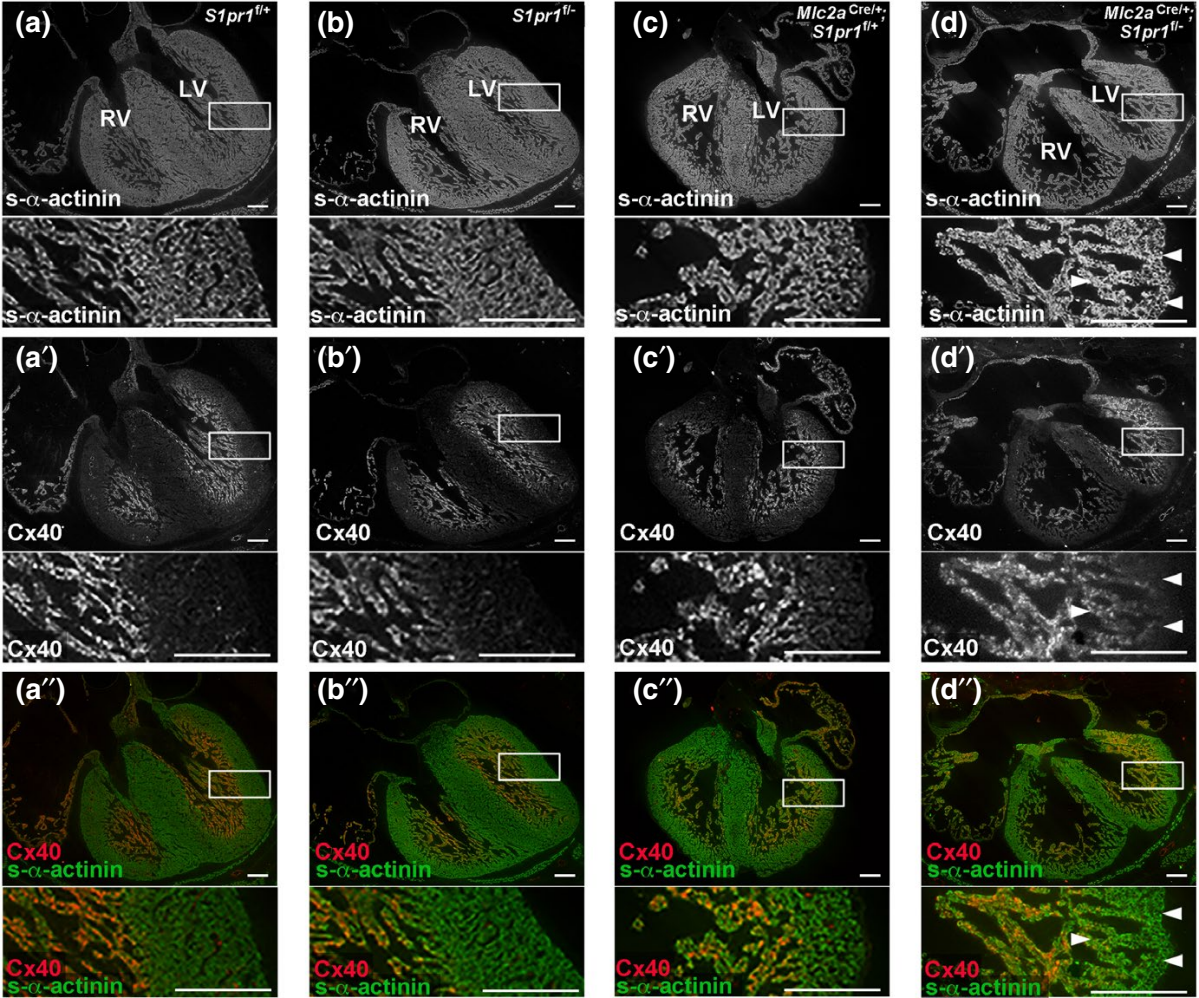
Connexin 40 (Cx40) expression pattern is disrupted in mice with embryonic cardiomyocyte S1pr1 deletion. Embryos were collected at 15.5 dpc. (a) *S1pr1* ^f/+^ embryonic heart. (b) *S1pr1* ^f/−^ embryonic heart. (c) *Mlc2a* ^Cre/+^; *S1pr1* ^f/+^ embryonic heart. (d) *Mlc2a* ^Cre/+^; *S1pr1* ^f/−^ mutant embryonic heart. (a–d) Immunostaining for s‐α‐actinin to mark all cardiomyocytes. (a′–d′) Immunostaining for Cx40. (a′′–d′′) Merged images for s‐α‐actinin and Cx40. Boxes indicate magnified region shown below each low‐power image. Arrows highlight regions of reduced Cx40 expression in trabecular myocardium adjacent to the thin compact layer in the mutant embryonic heart. Representative images from *n* = 3–6 per genotype are shown. LV, left ventricle. RV, right ventricle. Scale bar, 200 µm

During heart development, Purkinje fibers originate from trabecular myocardium. Like other components of the VCS, Purkinje fibers highly express Cx40 (encoded by *Gja5*). Furthermore, altered expression of Cx40 and Cx43 has been observed in mouse and human cardiomyopathies with aberrant ventricular conduction (Akar et al., 2004; Mueller et al., 2011; Sedmera et al., 2016; Tang et al., 2013). To investigate this potential mechanism of abnormal ventricular conduction in cardiomyocyte *S1pr1* mutant mice, we first assessed Cx40 expression in hearts from mice at 15.5 dpc. As previously reported, we observed no significant differences from the expected numbers of 15.5 dpc embryos across the four genotypes in this study (Clay et al., 2016) (Table S1). Also as previously reported, s‐α‐actinin staining of embryonic hearts from the three control genotypes showed normal ventricular compaction (Figure 3a–c), with roughly equal thickness of the trabecular and compact myocardium; by contrast, embryonic hearts from *Mlc2a^Cre^* ^/+^; *S1pr1^f^* ^/−^ mutant mice displayed deep trabeculations and a thin compact wall (Figure 3d). As expected, Cx40 was expressed in atria and trabecular myocardium in the three control genotypes (Figure 3a’–c’). In *Mlc2a^Cre^* ^/+^; *S1pr1^f^* ^/−^ mutant hearts, we predicted that all trabecular myocardium would express Cx40 at a high level. However, Cx40 expression was not uniformly expressed in the trabecular myocardium of *Mlc2a^Cre^* ^/+^; *S1pr1^f^* ^/−^ mutant hearts; trabecular myocardium closer to the thin compact wall expressed lower levels of Cx40 by immunostaining (Figure 3d’). We observed this Cx40 expression pattern for all six mutant embryonic hearts used for immunostaining (Figure S2). This Cx40 expression pattern has been noted in a subset of ventricular noncompaction models and has been termed “intermediate myocardium” (D'Amato et al., 2016; Rhee et al., 2018). We observed no significant differences in the immunostaining patterns for Cx43 or N‐Cadherin (Figure S3a,b).

**FIGURE 4:**
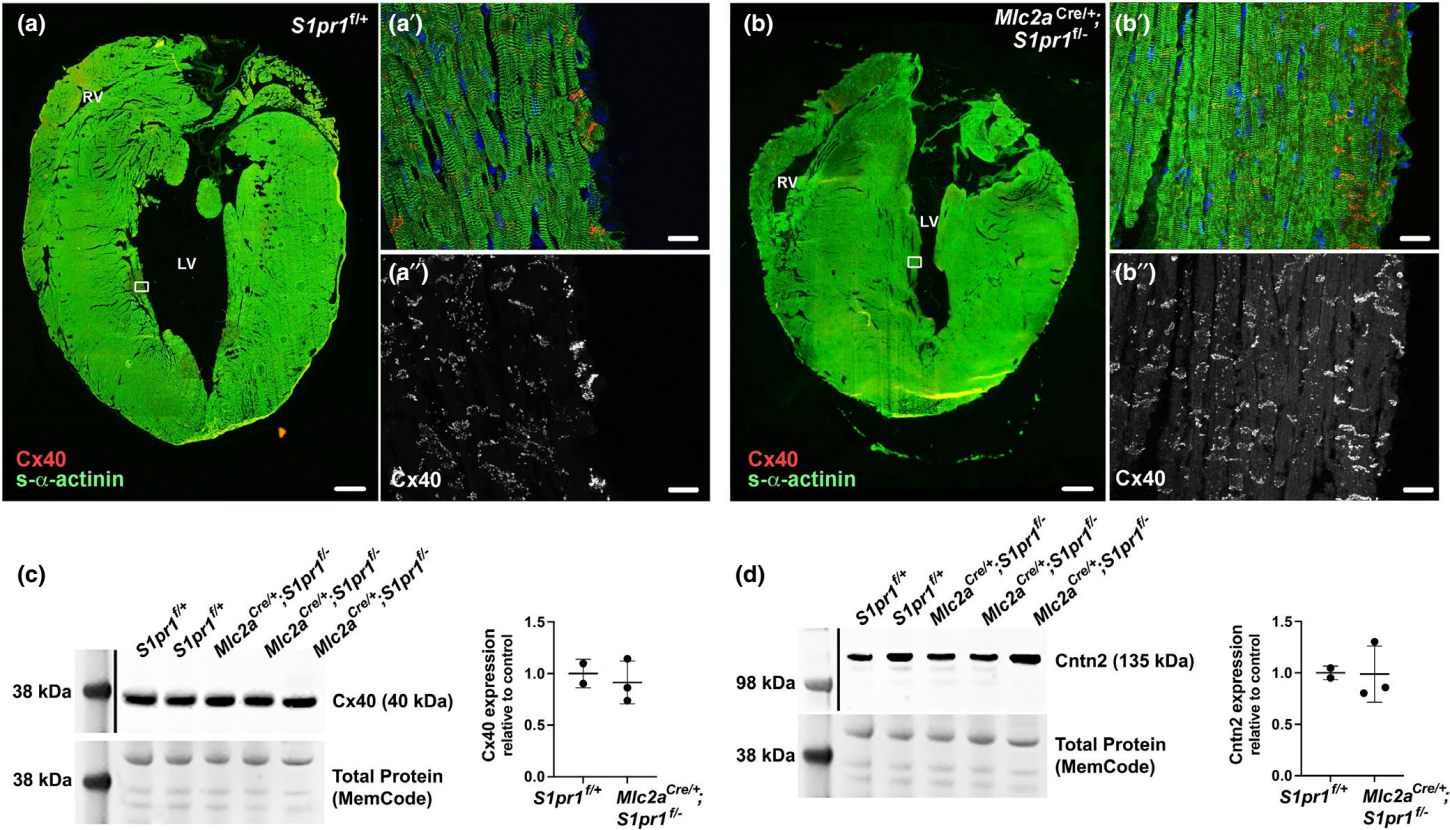
Normal markers of the ventricular conduction system in mice with embryonic cardiomyocyte S1pr1 deletion. Hearts from 12‐month‐old mice were perfused with cardioplegia solution, snap frozen, cryosectioned, and immunostained for s‐α‐actinin and Cx40. Representative images from *n* = 3–6 mice per genotype are shown. (a) *S1pr1* ^f/+^ control heart tiled imaged to show entire section. Scale bar, 500 µm. (a′) High‐power image from boxed area in panel (a). Note Cx40 staining at intercalated disks of VCS cardiomyocytes. Scale bar, 20 µm. (a′′) Cx40 staining alone from the high‐power image. (b) *Mlc2a* ^Cre/+^; *S1pr1* ^f/−^ mutant heart tiled image. Scale bar, 500 µm. (b′) High‐power image from boxed area in panel (b). Scale bar, 20 µm. (b′′) Cx40 staining alone from the high‐power image. (c) Immunoblot of lysates from whole left and right ventricles from 4‐month‐old mice shows no difference in Cx40 levels between *S1pr1* ^f/+^ control and *Mlc2a* ^Cre/+^; *S1pr1* ^f/−^ mutant hearts. For densitometry, Cx40 was normalized to the ~45 kDa band in the total protein blot for each lane, and values are presented as relative to the control average. *p* = 0.6515 by Student's *t*‐test. (d) Immunoblot of lysates from whole left and right ventricles from 4‐month‐old mice shows no difference in Contactin 2 (Cntn2) levels between *S1pr1* ^f/+^ control and *Mlc2a* ^Cre/+^; *S1pr1* ^f/−^ mutant hearts. For densitometry, Cntn2 was normalized to the ~45 kDa band in the total protein blot for each lane, and values are presented as relative to the control average. *p* = 0.9633 by Student's *t*‐test

Next, we examined Cx40 expression patterns in adult mice. Unlike the abnormal Cx40 expression pattern in embryonic *Mlc2a^Cre^* ^/+^; *S1pr1^f^* ^/−^ mutant hearts, adult mutant hearts demonstrated a Cx40 expression pattern that was similar to controls and restricted to cardiomyocytes within the bundle branches and Purkinje fibers (Figure 4; of note, the heart shown in Figure 4b is the same as in Figure 1d). We did not observe an increase of Cx40 immunostaining in *Mlc2a^Cre^* ^/+^; *S1pr1^f^* ^/−^ mutant hearts as compared with controls (Figure 4b). To quantitatively assess Cx40 expression, we collected whole hearts and isolated protein from combined left and right ventricles for immunoblotting. We did not detect a significant difference in Cx40 levels in ventricular lysates between *S1pr1* ^f/+^ and *Mlc2a^Cre^* ^/+^; *S1pr1^f^* ^/−^ mutant mice (Figure 4c). Because Cx40 is also expressed in coronary artery endothelium, we further assessed expression of Contactin‐2 (Cntn2), a cell adhesion molecule expressed specifically in postnatal VCS cardiomyocytes (Pallante et al., 2010). Again, we observed no differences in ventricular Cntn2 expression levels between control and mutant mice (Figure 4d). Finally, we assessed Cx43 and NCad expression via immunofluorescence, and similar patterns and intensities were noted across genotypes (Figure S4). Together, these results suggest that the noncompaction and abnormal Cx40 expression pattern in *Mlc2a^Cre^* ^/+^; *S1pr1^f^* ^/−^ mutant embryos may not disrupt development and maintenance of the VCS at later stages.

### 3.4 Cardiomyocyte *S1pr1* mutants display abnormal increased cardiac fibrosis

**FIGURE 5:**
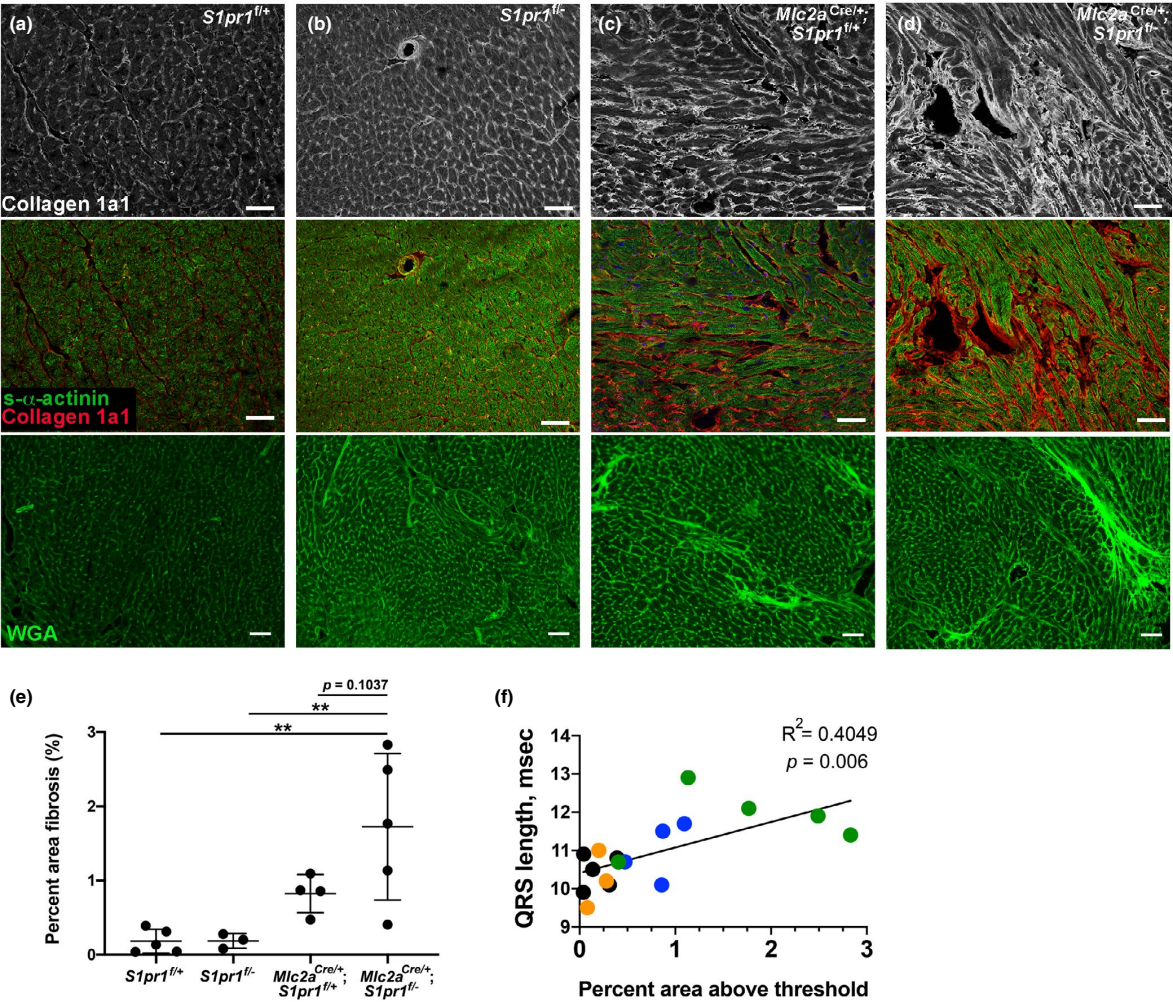
Cardiomyocyte S1pr1 mutants display increased fibrosis markers. (a–d) Hearts from 12‐month‐old mice were perfused with cardioplegia solution, snap frozen, cryosectioned, and immunostained. (a) *S1pr1* ^f/+^ heart. (b) *S1pr1* ^f/−^ heart. (c) *Mlc2a* ^Cre/+^; *S1pr1* ^f/+^ heart. (d) *Mlc2a* ^Cre/+^; *S1pr1* ^f/−^ mutant heart. Top row, Collagen 1a1 (Col1a1) and middle row, merged Col1a1 and s‐α‐actinin immunostaining from the same images. Bottom row, wheat germ agglutinin (WGA)‐Alexa Fluor 488 staining from different sections of the same hearts shown in the top and middle rows. Scale bar, 50 µm. (e) Percent area fibrosis within sections from 6‐ to 12‐month‐old mice was determined by measuring the area of interstitial WGA signal within the left ventricular myocardium of the heart section. *n* = 3–5 per genotype. **p < 0.01 by one‐way ANOVA with Bonferroni post‐hoc test for multiple comparisons. (f) Correlation between percent area fibrosis and QRS duration. Black points represent *S1pr1* ^f/+^ mice, orange points represent *S1pr1* ^f/−^ mice, blue points represent *Mlc2a* ^Cre/+^; *S1pr1* ^f/+^ mice, and green points represent *Mlc2a* ^Cre/+^; *S1pr1* ^f/−^ mice. Pearson's *r* correlation coefficient = 0.6363, *R* ^2^ = 0.4049, and two‐tailed *p* = 0.006

Structural abnormalities of the working myocardium, such as cardiac fibrosis, can also disrupt ventricular conduction. To investigate cardiac fibrosis as a potential mechanism for QRS prolongation in cardiomyocyte *S1pr1* mutants, we assessed heart sections using Collagen 1a1 (Col1a1) immunostaining and WGA‐Alexa Fluor 488 staining. We detected low levels of Col1a1 and WGA in heart sections from *S1pr1* ^f/+^ and *S1pr1* ^f/−^ mice, while sections from *Mlc2a^Cre^* ^/+^; *S1pr1^f^* ^/+^ mutant showed moderate levels of Col1a1 immunostaining (Figure 5a–c). Heart sections from *Mlc2a^Cre^* ^/+^; *S1pr1^f^* ^/−^ mutant mice displayed the highest level of Col1a1 signal (Figure 5d). We measured the percent area fibrosis within the heart sections and found significantly higher signal in *Mlc2a^Cre^* ^/+^; *S1pr1^f^* ^/−^ mutant hearts as compared with *S1pr1* ^f/+^ and *S1pr1* ^f/−^ mice (Figure 5e, Figure S5). As we observed with QRS duration, the difference in fibrosis area did not reach statistical significance between *Mlc2a^Cre^* ^/+^; *S1pr1^f^* ^/+^ hearts and *Mlc2a^Cre^* ^/+^; *S1pr1^f^* ^/−^ mutant hearts. However, we found a statistically significant correlation between QRS duration and percent area fibrosis (Figure 5f). These results demonstrate that cardiomyocyte‐specific loss of *S1pr1* increases cardiac fibrosis in surviving adult mice, and that this increased cardiac fibrosis correlates with abnormal ventricular conduction in these mutant mice.

## 4 DISCUSSION

Here, we report the phenotype of adult mice that survive embryonic deletion of *S1pr1* in cardiomyocytes. We observed cardiac hypertrabeculation consistent with ventricular noncompaction, preserved systolic function, and normal lifespan in surviving *Mlc2a^Cre^* ^/+^; *S1pr1^f^* ^/−^ mutant mice. Cardiomyocyte *S1pr1* mutant mice also demonstrated conduction abnormalities with prolonged QRS in the awake and active state. The conduction system marker Cx40 displayed an abnormal expression pattern in *Mlc2a^Cre^* ^/+^; *S1pr1^f^* ^/−^ mutant embryos. However, Cx40 and Cntn2 expression in adult mice did not differ from littermate controls, which suggests normalized development and maintenance of the VCS in cardiomyocyte *S1pr1* mutant mice. Finally, cardiomyocyte *S1pr1* mutant mice demonstrated increased cardiac fibrosis that correlated with QRS prolongation.

Purkinje cells are thought to arise from trabecular cardiomyocytes (Viragh & Challice, 1982), and conduction system development in the setting of persistent trabeculation and noncompaction is not well understood. In mammals, the cardiac conduction system is specified early in cardiac development. Fate mapping studies in Cx40‐positive cardiomyocytes showed that VCS progenitor cells differentiate into both mature conductive cells and working myocardial cells prior to 14.5 dpc, but that by 16.5 dpc the conductive fate is established in Cx40‐positive VCS progenitor cells (Miquerol et al., 2010). Experiments in *Cx40*‐eGFP reporter mice demonstrated that *Nkx2*.*5* haploinsufficiency disrupted postnatal maturation of the Purkinje fiber network; furthermore, embryonic deletion of *Nkx2*.*5* in trabecular myocardium using *Cx40*‐*CreERT2* led to hypertrabeculation, Purkinje fiber hypoplasia, subendocardial fibrosis, and conduction abnormalities in adult hearts (Choquet et al., 2018; Meysen et al., 2007). Of note, the Cx40‐eGFP expression pattern was normal in hearts from *Cx40*‐*eGFP*; *Nkx2*.*5* ^+/−^ embryos at 16.5 dpc, while Purkinje fiber morphology was disrupted at birth in *Cx40*‐*eGFP*; *Nkx2*.*5* ^+/−^ hearts (Meysen et al., 2007). By contrast, our results in 15.5 dpc *Mlc2a^Cre^* ^/+^; *S1pr1^f^* ^/−^ mutant embryos revealed that trabecular myocardium did not uniformly express Cx40, with lower expression in the region adjacent to the thin compact myocardium (Figure 3d). However, we did not observe differences in Cx40 expression pattern or level in adult *Mlc2a^Cre^* ^/+^; *S1pr1^f^* ^/−^ mutant hearts. Therefore, while this region of reduced Cx40 trabecular myocardium may contribute to abnormal compaction, the altered Cx40 expression in that region does not appear to affect further maturation of the VCS in adult mice. It is possible that surviving *Mlc2a^Cre^* ^/+^; *S1pr1^f^* ^/−^ mutant mice did not have the region of reduced Cx40 expression during their development; however, the presence of altered Cx40 expression in 100% of stained mutant embryonic hearts (Figure S2) argues against this possibility. Future studies with *Cx40*‐*eGFP* or *Cntn2*‐*EGFP* lines will more definitively assess bundle branch density in *Mlc2a^Cre^* ^/+^; *S1pr1^f^* ^/−^ mutant hearts.

Other mouse models of ventricular noncompaction have demonstrated non‐uniform expression of Cx40 in the trabecular myocardium; specifically, disruption of some components of the Notch signaling pathway (combined deletion of cardiomyocyte *Jagged1 and Jagged2*, as well as endothelial overexpression of *Manic Fringe)* and endothelial‐specific deletion of *Ino80* led to the same Cx40 expression pattern that we observed (Luxán et al., 2013; Rhee et al., 2018). This “intermediate myocardium” represents a margin between trabecular and compact myocardium, with cardiomyocytes that display trabecular morphology but express molecular markers of compact/non‐trabecular myocardium. *S1pr1* was not reported to be differentially expressed in the other models that display intermediate myocardium, and whether S1P_1_ signaling pathways intersect with Notch signaling or Ino80‐mediated chromatin remodeling in developing cardiomyocytes remains to be determined.

In adult mice, we observed an intermediate increase in QRS prolongation and cardiac fibrosis in *Mlc2a^Cre^* ^/+^; *S1pr1^f^* ^/+^ mice, as compared with Cre‐negative controls and *Mlc2a^Cre^* ^/+^; *S1pr1^f^* ^/−^ mutant mice. These results suggest a potential interaction between *S1pr1* dosage and the presence of *Mlc2a^Cre^* ^/+^. Heterozygosity for *S1pr1* did not lead to a change in either QRS duration or cardiac fibrosis, which indicates that *S1pr1* dosage alone was not the primary driver of these phenotypes. *Mlc2a* is expressed in both atrial and ventricular cardiomyocytes up to embryonic day 11 and then restricted to atrial cardiomyocytes by embryonic day 12 in mice (Kubalak et al., 1994). *Mlc2a* heterozygous mice survive with no obvious cardiac phenotype (Huang et al., 2003), so it was predicted that haploinsufficiency would not affect cardiac development. However, *Mlc2a* null mice did not survive beyond 11.5 dpc due to severely reduced atrial contractility and failure to progress through normal ventricular trabeculation and compaction (Huang et al., 2003). It is possible that haploinsufficiency for *Mlc2a* in the *Mlc2a^Cre^* ^/+^ background could lead to subtle but functionally important reduction in atrial contractility; in such a setting, *Mlc2a* haploinsufficiency might create a sensitized background in which *S1pr1* gene dosage could contribute to the phenotype. Specifically, *Mlc2a* haploinsufficiency and *S1pr1* heterozygosity in cardiomyocytes may lead to the intermediate phenotype in *Mlc2a^Cre^* ^/+^; *S1pr1^f^* ^/+^ mice, while *Mlc2a* haploinsufficiency and loss of *S1pr1* in cardiomyocytes drives the full noncompaction, QRS prolongation, and cardiac fibrosis phenotypes. We cannot rule out the possibility that cardiomyocyte‐specific deletion of *S1pr1* may also require *Mlc2a* haploinsufficiency for the noncompaction phenotype. Because *Mlc2a* is not expressed in ventricular cardiomyocytes after 12 dpc, it is unlikely that toxicity from accumulated Cre makes a large contribution to these phenotypes.

In terms of genetic cardiomyopathies, pathogenic *S1PR1* variants have not been described in humans. However, the Genome Aggregation Database (gnomAD), a data set of 125,748 exome sequences, and 15,708 whole‐genome sequences from unrelated individuals, identified two unique frameshift variant alleles at amino acid positions 255 and 256 (rs1166435525 and rs1395550411 in the dbSNP database, respectively) (Holmes et al., 2019; Lek et al., 2016). These two variants disrupt S1P_1_ at the origin of transmembrane domain six. One stop‐gained variant allele at amino acid position 283 was observed as well (rs760714388 in dbSNP). Furthermore, missense variants identified in the NHLBI Exome Sequencing Project have been functionally assessed: the Arg120Pro variant (rs149198314; identified in 1/13,005 alleles) failed to activate S1P_1_ signaling or internalization in response to S1P, and Arg120 was found to be critical for S1P binding (ESP; Obinata et al., 2014; Parrill et al., 2000). Hence, individuals who are heterozygous for predicted loss‐of‐function *S1PR1* alleles have been observed at very low frequency, and cardiomyopathy exome/genome sequencing studies may reveal additional *S1PR1* variants that disrupt S1P1 function.

Cardiac fibrosis has been associated with conduction abnormalities, including QRS prolongation, presumably due to the disruption of cardiomyocyte–cardiomyocyte electrical coupling and late depolarization in regions of fibrosis (Nazarian et al., 2010; Nguyen et al., 1988). Given the observation of both intermediate fibrosis and intermediate QRS prolongation in *Mlc2a^Cre^* ^/+^; *S1pr1^f^* ^/+^ mice that have no hypertrabeculation, we hypothesize that *Mlc2a* haploinsufficiency and *S1pr1* heterozygosity in cardiomyocytes can lower the threshold for cardiac fibrosis, possibly due to abnormal cardiac mechanics. The hypertrabeculation in *Mlc2a^Cre^* ^/+^; *S1pr1^f^* ^/−^ mutant mice may further impair cardiac mechanics and lead to increased fibrosis. This increase in fibrosis is the likely etiology of QRS prolongation in cardiomyocyte *S1pr1* mutant mice. Alternatively, hypertrabeculation itself may contribute to QRS prolongation, although trabecular length correlation with QRS length (*R* ^2^ = 0.2414, *p* = 0.04) was less strong than the correlation between fibrosis and QRS length (*R* ^2^ = 0.4049, *p* = 0.006; Figure 5c). In endothelial cells, S1P_1_ contributes to mechanosensing and endothelial cell alignment (Jung et al., 2012); future experiments will investigate possible roles for S1P_1_ in cardiomyocyte mechanosensation pathways.

The source of S1P that activates cardiomyocyte S1P_1_ is not definitively known and potentially includes endocrine, paracrine, and autocrine sources. We found no phenotype in mice that lacked *Sphk1* and *Sphk2* in cardiomyocytes of the developing heart, which argues against autocrine S1P mechanism in cardiomyocytes and suggests that cardiomyocyte‐specific generation of S1P is not necessary for normal cardiac development. Recent studies of Nogo‐B, a protein that inhibits the rate‐limiting enzyme in sphingolipid biosynthesis, suggest endothelial cells as a very likely origin (Zhang et al., 2016).

In conclusion, we report that mice that survive embryonic deletion of *S1pr1* in cardiomyocytes show hypertrabeculation with normal lifespan and normal systolic function, prolonged QRS duration, and increased cardiac fibrosis. Our results suggest an interaction between *Mlc2a* haploinsufficiency (*Mlc2a* ^Cre/+^) and *S1pr1* dosage in cardiomyocytes. Furthermore, few adult mouse models for hypertrabeculation/noncompaction exist, and *Mlc2a^Cre^* ^/+^; *S1pr1^f^* ^/−^ mutant mice represent a useful model to investigate this cardiomyopathy. The presence of conduction abnormalities in hypertrabeculated hearts, despite having normal systolic function, may be of clinical relevance for patients with hypertrabeculation.

## DISCLOSURES

This work was supported in part by a grant from the Gilead Sciences Research Scholars Program in Cardiovascular Disease (L.D.W.). The funding source had no role in the design, collection and analysis of data, writing, or submission of the manuscript.

## AUTHOR CONTRIBUTIONS

R.J., M.K., and L.D.W. conceived and designed the research; R.J., M.K., J.W., D.F.L., B.L., and L.D.W. performed the experiments; R.J., M.K., D.F.L., and L.D.W. analyzed the data; R.J. and L.D.W. interpreted the results of experiments; R.J. and L.D.W. drafted the manuscript; R.J., M.K., J.W., D.F.L., B.L., J.C., and L.D.W. edited and revised the manuscript; R.J., M.K., J.W., D.F.L., B.L., J.C., and L.D.W. approved the final version of the manuscript.

## Supporting information



Tables S1–S3, Figures S1–S5
