# Supplementary material for: Deletion of Sphingosine 1‐Phosphate receptor 1 in cardiomyocytes during development leads to abnormal ventricular conduction and fibrosis
Source: Physiol Rep. 2021 Oct 7;9(19):e15060. doi: 10.14814/phy2.15060 (PMC8496155; doi:10.14814/phy2.15060)
Supplement: Supplementary file 1 — Tables S1–S3, Figures S1–S5 [file PHY2-9-e15060-s001.docx]

**SUPPLEMENTAL TABLE 1**

|  | ***Mlc2a*^+/+^;**  ***S1pr1*^f/+^** | ***Mlc2a^+^*^/+^;**  ***S1pr1*^f/-^** | ***Mlc2a*^Cre/+^;**  ***S1pr1*^f/+^** | ***Mlc2a*^Cre/+^;**  ***S1pr1*^f/-^** |
| --- | --- | --- | --- | --- |
| **Weaning** | 53 (40.5) | 48 (40.5) | 43 (40.5) | 18 (40.5) |
| **15.5 dpc** | 15 (13.25) | 18 (13.25) | 10 (13.25) | 10 (13.25) |

**Supplemental Table 1.** Reduced survival to weaning in Mlc2a^Cre/+^; *S1pr1*^f/-^ mutant mice. Genotypes are shown for pups from Mlc2a^Cre/+^; *S1pr1*^+/-^ males crossed with *S1pr1*^f/f^ females. The observed number for each genotype is followed by the expected number in parentheses. At weaning, only 44% of the expected numbers of Mlc2a^Cre/+^; *S1pr1*^f/-^ mutant mice were observed (χ^2^ 17.9, p = 0.0005). At 15.5 dpc, there were no significant differences between observed and expected numbers across the four genotypes (χ^2^ 3.825, p = 0.3171).

**SUPPLEMENTAL TABLE 2**

|  | ***Mlc2a*^+/+^; *S1pr1*^f/+^** | ***Mlc2a*^+/+^; *S1pr1*^f/-^** | ***Mlc2a*^Cre/+^; *S1pr1*^f/+^** | ***Mlc2a*^Cre/+^; *S1pr1*^f/-^** |
| --- | --- | --- | --- | --- |
| PSAX IVS;d (mm) | 1.11 +/- 0.18 | 1.12 +/- 0.17 | 1.06 +/- 0.12 | 1.14 +/- 0.13 |
| PSAX IVS;s (mm) | 1.51 +/- 0.21 | 1.41 +/- 0.14 | 1.61 +/- 0.17 | 1.66 +/- 0.18 |
| PSAX LVID;d (mm) | 3.85 +/- 0.52 | 3.68 +/- 0.46 | 3.76 +/- 0.40 | 3.39 +/- 0.24 |
| PSAX LVID;s (mm) | 2.70 +/- 0.75 | 2.49 +/- 0.45 | 2.31 +/- 0.48 | 2.05 +/- 0.37 |
| PSAX LVPW;d (mm) | 0.87 +/- 0.16 | 0.91 +/- 0.02 | 0.97 +/- 0.10 | 1.34 +/- 0.31 |
| PSAX LVPW;s (mm) | 1.17 +/- 0.17 | 1.21 +/- 0.01 | 1.44 +/- 0.15 | 1.70 +/- 0.43 |
| PSAX FS (%) | 31.01 +/- 12.19 | 24.74 +/- 1.93 | 38.95 +/- 7.01 | 39.63 +/- 10.19 |
| PSAX EF (%) | 57.64 +/- 17.86 | 49.5 +/- 2.79 | 69.63 +/- 8.55 | 10.11 +/- 12.23 |
|  |  |  |  |  |
| PSLAX IVS;d (mm) | 1.02 +/- 0.21 | 1.01 +/- 0.17 | 1.02 +/- 0.24 | 1.17 +/- 0.23 |
| PSLAX IVS;s (mm) | 1.32 +/- 0.27 | 1.38 +/- 0.28 | 1.47 +/- 0.32 | 1.68 +/- 0.28 |
| PSLAX LVID;d (mm) | 3.91 +/- 0.30 | 3.85 +/- 0.41 | 3.99 +/- 0.35 | 3.47+/-0.24 |
| PSLAX LVID;s (mm) | 2.92 +/- 0.22 | 2.75 +/- 0.49 | 2.81 +/- 0.44 | 2.27+/-0.45 |
| PLSAX LVPW;d (mm) | 0.94 +/- 0.16 | 0.79 +/- 0.07 | 0.82 +/- 0.17 | 1.08 +/- 0.26 |
| PLSAX LVPW;s(mm) | 1.19 +/- 0.17 | 1.06 +/- 0.15 | 1.07 +/- 0.23 | 1.44 +/- 0.26 |
| PSLAX FS (%) | 25.20 +/- 4.18 | 28.84 +/- 7.24 | 29.76 +/- 6.41 | 34.94 +/- 10.14 |
| PSLAX EF (%) | 50.20 +/- 6.90 | 55.70 +/- 10.85 | 57.01 +/- 9.24 | 64.09 +/- 13.47 |
|  |  |  |  |  |
| Trabecular, mm | 0.60 +/- 0.13 | 0.57 +/- 0.15 | 0.68 +/- 0.21 | 1.12 +/- 0.19 |
| Compact, mm | 0.79 +/- 0.20 | 0.57 +/- 0.09 | 0.85 +/- 0.19 | 0.82 +/- 0.10 |
| Trabecular:Compact ratio | 0.79 +/- 0.14 | 0.99 +/- 0.17 | 0.83 +/- 0.23 | 1.40 +/- 0.28 |

**Supplemental Table 2.** Echocardiographic data from Mlc2a^Cre/+^; *S1pr1* mice. Values are averages +/- standard deviation. PSAX: parasternal short axis. PSLAX: parasternal long axis. IVS;d: interventricular septum thickness in diastole. IVS;s: interventricular septum thickness in systole. LVID;d: left ventricular internal dimension in diastole. LVID;s: left ventricular internal dimension in systole. LVPW;d: left ventricular posterior wall thickness in diastole. LVPW;s: left ventricular posterior wall thickness in systole. FS: fractional shortening. EF: ejection fraction. Trabecular: average trabecular myocardium thickness. Compact: average compact myocardium thickness.

**SUPPLEMENTAL TABLE 3**

|  | ***Mlc2a*^+/+^;**  ***Sphk1*^f/+^;**  ***Sphk2^-/-^*** | ***Mlc2a*^+/+^;**  ***Sphk1*^f/-^;**  ***Sphk2^-/-^*** | ***Mlc2a^Cre^*^/+^;**  ***Sphk1*^f/+^;**  ***Sphk2^-/-^*** | ***Mlc2a^Cre^*^/+^;**  ***Sphk1*^f/-^;**  ***Sphk2^-/-^*** |
| --- | --- | --- | --- | --- |
| **Weaning** | 14 (9) | 9 (9) | 5 (9) | 8 (9) |

**Supplemental Table 3.** Generation of S1P ligand in cardiomyocytes is not required for survival. Genotypes are shown for pups from Mlc2a^Cre/+^; *Sphk1*^+/-^; *Sphk2*^-/-^ mice crossed with *Sphk1*^f/f^; *Sphk2*^-/-^ mice. The observed number of weaned mice for each genotype is followed by the expected number of weaned mice in parentheses. There were no significant differences between observed and expected numbers across the four genotypes (χ^2^ 4.556, p = 0.1979).

**SUPPLEMENTAL FIGURE 1**

**
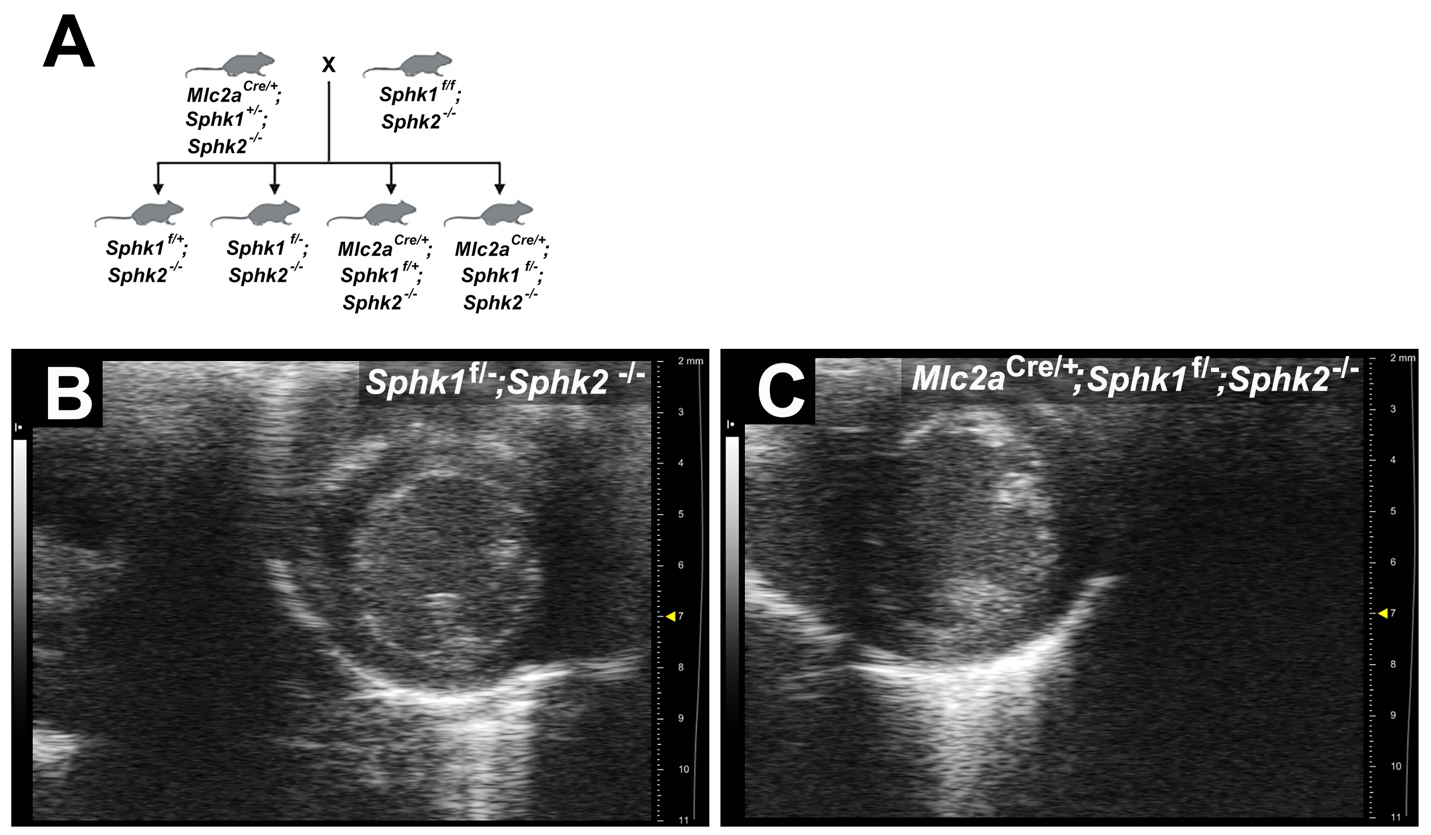
­­­**

**Supplemental Figure 1.** **Generation of S1P ligand in cardiomyocytes is not required for normal heart development.** A) Breeding strategy. B) Parasternal short axis view from a 5-month old *Sphk1*^f/-^; *Sphk2*^-/-^ control mouse. C) Parasternal short axis view from an Mlc2a^Cre/+^; *Sphk1*^f/-^; *Sphk2*^-/-^ littermate mutant mouse. Note the absence of hypertrabeculated myocardium in the Mlc2a^Cre/+^; *Sphk1*^f/-^; *Sphk2*^-/-^ mutant heart.

**SUPPLEMENTAL FIGURE 2**

**
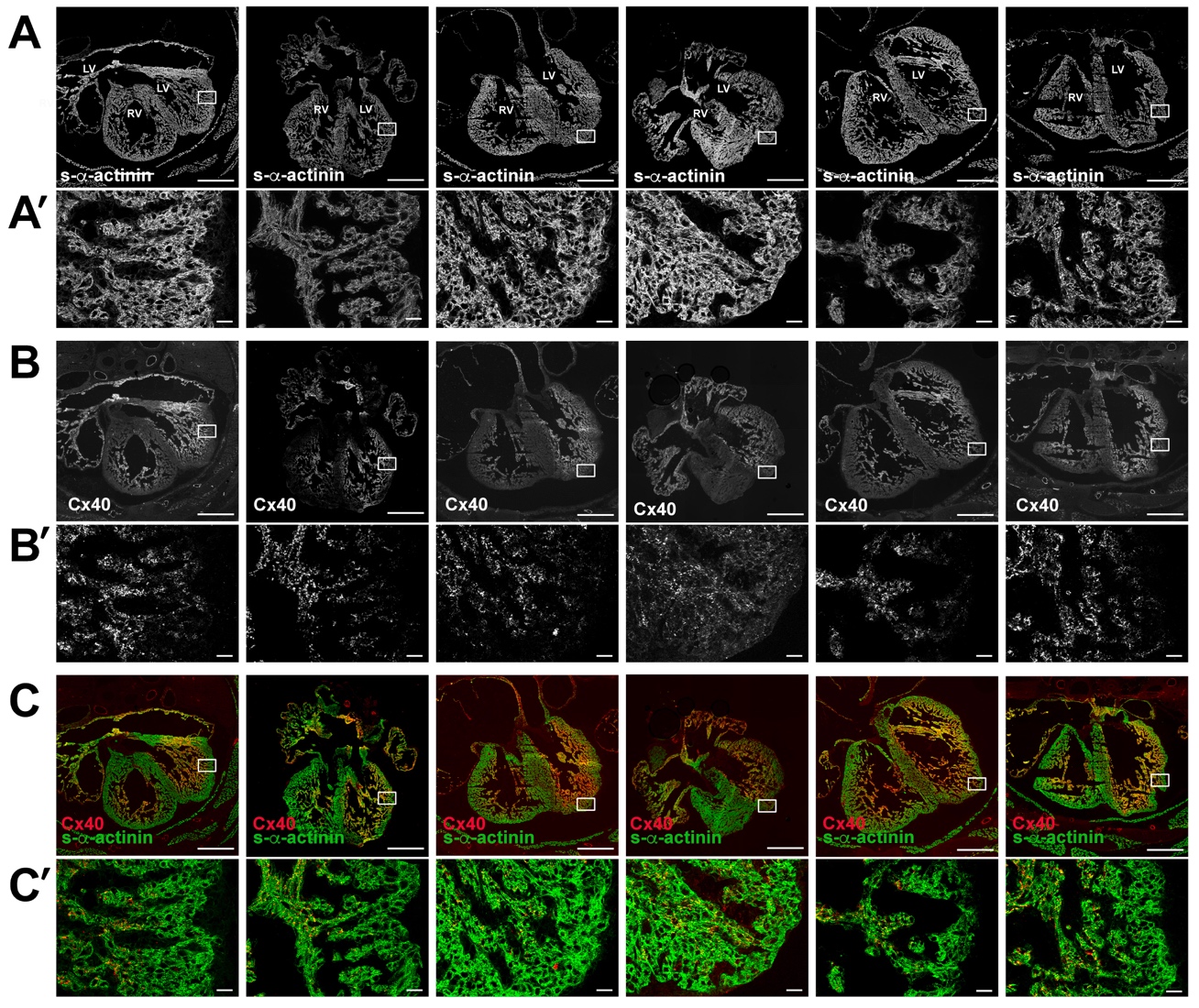
**

**Supplemental Figure 2. s-α-actinin and** **Connexin 40 immunostaining in hearts from six *Mlc2a^Cre/+^; S1pr1^f/-^* mutant** **embryos collected at 15.5 dpc.** A) s-α-actinin immunostaining, full section. Scale bar = 500μm. LV, left ventricle. RV, right ventricle. A’) s-α-actinin immunostaining, high-magnification image from region indicated by the box in the image above. Scale bar = 20μm. Note the presence of thin compact wall and long trabeculae in all six embryonic hearts. B) Connexin 40 (Cx40) immunostaining, full section. Scale bar = 500μm. B’) Cx40 immunostaining, high-magnification image from region indicated by the box in the image above. Scale bar = 20μm. Note high-intensity staining in trabecular region closest to the ventricular space, and lower-intensity staining in the trabecular region closest to the thin compact wall. C) s-α-actinin and Cx40 immunostaining overlay, full section. Scale bar = 500μm. C’) s-α-actinin and Cx40 immunostaining overlay, high-magnification image from region indicated by the box in the image above. Scale bar = 20μm.

**SUPPLEMENTAL FIGURE 3**

**
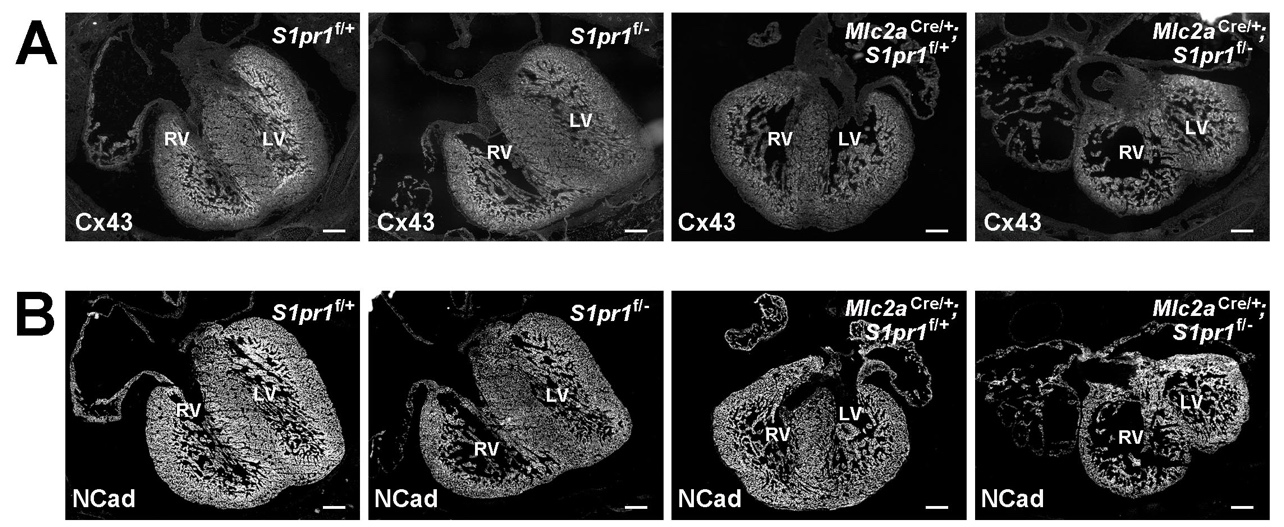
**

**Supplemental Figure 3. Connexin 43, and N-Cadherin immunostaining in hearts from embryos collected at 15.5 dpc.** A) Connexin 43 (Cx43) immunostaining in hearts from embryos of the indicated genotypes. Representative images from n = 3 per genotype are shown. No significant differences were noted among the four genotypes. Scale bar, 200 μm. B) N-cadherin (NCad) immunostaining in hearts from embryos of the indicated genotypes. Representative images from n = 3 per genotype are shown. No significant differences were noted among the four genotypes. Scale bar, 200 μm. LV, left ventricle. RV, right ventricle.

**SUPPLEMENTAL FIGURE 4**

**
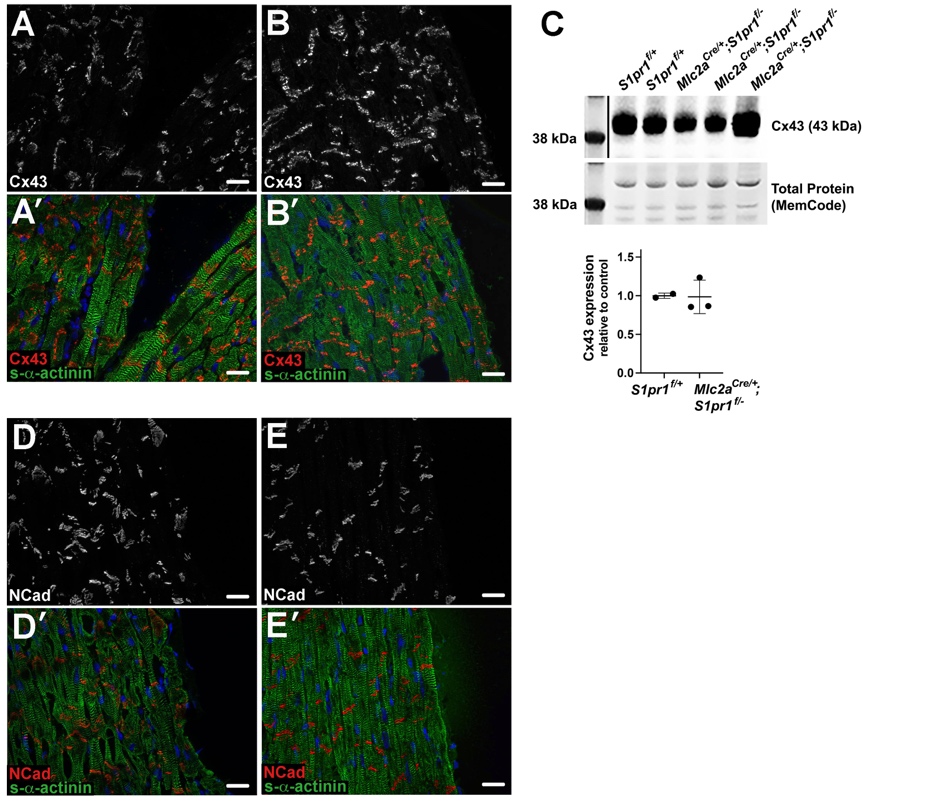
**

**Supplemental Figure 4. Normal expression of connexin 43 (Cx43) and N-Cadherin (NCad) in mice with embryonic cardiomyocyte *S1pr1* deletion.** Hearts from 12 month old mice were perfused with cardioplegia solution, snap frozen, cryosectioned, and immunostained as indicated. Representative images from n = 3-6 mice per genotype are shown.

A*) S1pr1*^f/+^ control heart immunostained for Cx43. A’) Merged immunostaining for s-α-actinin (myofibril marker) and Cx43 in the *S1pr1*^f/+^ control section shown in panel A.

B) *Mlc2a^Cre/+;^ S1pr1^f/-^* mutant heart immunostained for Cx43. B’) Merged immunostaining for s-α-actinin and Cx43 in the *S1pr1*^f/+^ *Mlc2a^Cre/+;^ S1pr1^f/-^* mutant section shown in panel B.

C) Immunoblot of lysates from whole left ventricle and right ventricle show no consistent difference in Cx43 levels between *S1pr1*^f/+^ control and *Mlc2a^Cre/+;^ S1pr1^f/-^* mutant hearts. For densitometry, Cx43 was normalized to the ~45kD band in the total protein blot for each lane, and values are presented as relative to the control average. P = 0.9358 by Student’s t test.

D) *S1pr1*^f/+^ control heart immunostained for NCad. D’) Merged immunostaining for s-α-actinin and NCad in the *S1pr1*^f/+^ control section shown in panel D.

E) *Mlc2a^Cre/+;^ S1pr1^f/-^* mutant heart immunostained for NCad. E’) Merged immunostaining for s-α-actinin and NCad in the *Mlc2a^Cre/+;^ S1pr1^f/-^* mutant section shown in panel E. No significant differences were noted among the genotypes. Scale bar, 20 μm.

**SUPPLEMENTAL FIGURE 5**

**
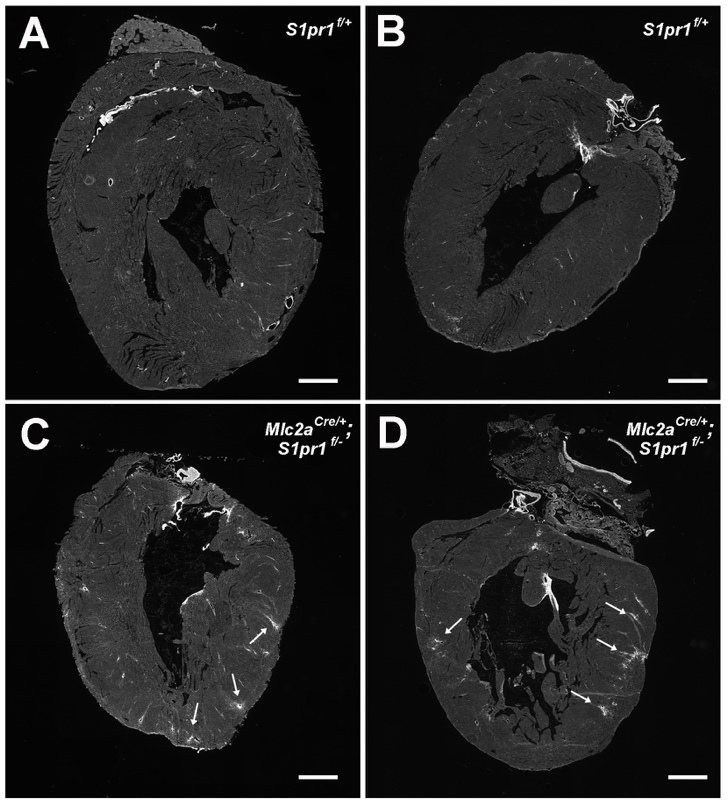
**

**Supplemental Figure 5. Increased cardiac fibrosis markers in cardiomyocyte *S1pr1* mutants.** Wheat germ agglutinin (WGA) Alexa Fluor488 staining in sections from adult hearts. A, B) *S1pr1^f+-^* control at 12 months of age. C) *Mlc2a^Cre/+;^ S1pr1^f/-^* mutant at 12 months of age. D) *Mlc2a^Cre/+;^ S1pr1^f/-^* mutant at 6 months of age. Arrows denote regions of interstitial fibrosis. Representative images from n = 3-6 per genotype are shown. Scale bar, 1mm.
